# 3D Spheroid Culture Enhances the Expression of Antifibrotic Factors in Human Adipose-Derived MSCs and Improves Their Therapeutic Effects on Hepatic Fibrosis

**DOI:** 10.1155/2016/4626073

**Published:** 2016-02-28

**Authors:** Xuan Zhang, Ming-Gen Hu, Ke Pan, Chong-Hui Li, Rong Liu

**Affiliations:** ^1^Department of Hepatobiliary and Pancreatic Surgical Oncology, Chinese PLA General Hospital and Chinese PLA Medical College, No. 28 Fuxing Road, Haidian District, Beijing 100853, China; ^2^Department and Institute of Hepatobiliary Surgery, Chinese PLA General Hospital and Chinese PLA Medical College, No. 28 Fuxing Road, Haidian District, Beijing 100853, China

## Abstract

Three-dimensional (3D) cell culture has been reported to increase the therapeutic potentials of mesenchymal stem cells (MSCs). However, the action mechanisms of 3D MSCs vary greatly and are far from being thoroughly investigated. In this study, we aimed to investigate the therapeutic effects of 3D spheroids of human adipose-derived MSCs for hepatic fibrosis. Our results showed that 3D culture enhanced the expression of antifibrotic factors by MSCs, including insulin growth factor 1 (IGF-1), interleukin-6 (IL-6), and hepatocyte growth factor (HGF).* In vitro* studies indicated conditioned medium of 3D cultured MSCs protected hepatocytes from cell injury and apoptosis more effectively compared with 2D cultured cells. More importantly, when transplanted into model mice with hepatic fibrosis, 3D spheroids of MSCs were more beneficial in ameliorating hepatic fibrosis and improving liver function than 2D cultured cells. Therefore, the 3D culture strategy improved the therapeutic effects of MSCs and might be promising for treatment of hepatic fibrosis.

## 1. Introduction

Hepatic fibrosis refers to the increased deposition of extracellular matrix (ECM) in the liver parenchyma and threatens public health globally [1]. Multiple stimuli, including viral infection, cholestasis, toxins, autoimmune disorders, and metabolic diseases, may induce hepatic injury and fibrosis, which possibly progresses to cirrhosis and finally leads to liver failure and death [2]. Organ transplantation remains the ultimate treatment strategy for end-stage liver disease. However, it is limited in clinical applications due to shortage of organ sources, complications of liver transplantation, recurrence of diseases, and expensive costs.

Cell-based therapy using mesenchymal stem cells (MSCs) has been reported to be a promising treatment strategy for hepatic fibrosis [3–6]. The therapeutic effects of MSCs are based on their potential to differentiate into hepatocytes, their immunomodulatory properties, and their paracrine secretions [7]. The paracrine effects act as an important mechanism. Systemic infusion of bone marrow MSC-conditioned medium could inhibit hepatocellular death and stimulate liver regeneration in a D-galactosamine-induced rat model of acute liver injury [8]. Another study demonstrated that human umbilical cord matrix stem cells enhanced endogenous liver generation through paracrine effects in mice with CCl_4_-induced acute liver failure [9]. Besides liver regeneration, MSCs have been reported to have antifibrotic properties by paracrine secretions of cytokines, such as insulin growth factor 1 (IGF-1), interleukin-6 (IL-6), and hepatocyte growth factor (HGF) [10].

Paracrine actions of MSCs are limited under regular culture conditions, which impairs the therapeutic effects. Recent studies indicated that three-dimensional (3D) culture promoted differentiation of MSCs [11, 12] and enhanced their therapeutic potentials [13]. Aggregation of MSCs into 3D spheroids increased the expression of the anti-inflammatory protein TNF*α* stimulated gene/protein 6 (TSG-6) [14], as well as their paracrine secretion of angiogenic factors, including vascular endothelial growth factor (VEGF), basic fibroblast growth factor (bFGF), and angiogenin [15]. 3D spheroids of MSCs have been shown to exert therapeutic benefits on several diseases, including peritonitis [14], kidney injury [16], and myocardial infarction [17]. However, the action mechanisms of 3D MSCs varied greatly; for example, enhanced anti-inflammatory properties in peritonitis were mainly due to unregulated secretion of TGS-6 [14]; improvement of ischemic kidney injury was related to the promotion of angiogenesis [16], while enhanced myocardial repair was related to the increased engraftment and differentiation of transplanted cells [17]. Hepatic fibrosis is different from tissue damage in which the therapeutic effects of 3D MSCs have been investigated. Therefore, it remains to be investigated whether the 3D culture strategy is helpful to MSC-based therapy of hepatic fibrosis.

In this study, human adipose-derived MSCs were isolated and cultured as 3D spheroids. We first studied the protection effects of 3D spheroid-derived MSCs on injured murine hepatocytes* in vitro*. To further investigate the therapeutic potentials of 3D spheroids* in vivo*, 3D spheroid-derived MSCs were transplanted into the hepatic fibrosis model mice. The hepatic fibrosis and liver function were thereafter evaluated.

## 2. Methods and Materials

### 2.1. Isolation and Cultivation of Human Adipose-Derived MSCs

The study was approved by the hospital ethics committee. Adipose tissues were isolated from raw human lipoaspirates, washed with phosphate buffer solution (PBS), and cut into pieces with scissors. The tissues were then digested with 0.1% collagenase I (Sigma) and 0.1% trypsin (Sigma) for 30 min, which was terminated by adding equal volume of *α*-MEM medium (Gibco) containing 10% fetal bovine serum (FBS, Gibco). After filtering with 80 *μ*m meshes, the solution was centrifuged at 1500 rpm for 10 min. The cell pellet was resuspended in fresh medium (*α*-MEM/10% FBS) and seeded in tissue culture plates. Cells were cultured in an incubator at 37°C with 5% CO_2_ and passaged when they reached 90% confluence, using 0.25% trypsin at a 1 : 3 split ratio.

### 2.2. Generation and Dissociation of 3D Spheroids of MSCs

Human adipose-derived MSCs of P3–P6 with good growth status were collected by trypsin digestion, and 3D spheroids were generated according to the previous report [14]. Briefly, MSCs were prepared as 7.5 × 10^5^/mL cell suspensions. 35 *μ*L of cell solution per drop (containing about 25,000 cells) was prepared onto the covers of culture plate. Cell drops were cultured inversely for 3 days in an incubator at 37°C with 5% CO_2_. To obtain 3D spheroid-derived cells, the spheroids were digested with 0.05% trypsin/EDTA for 5–10 min, which was terminated by adding equal volume of fresh medium (*α*-MEM/10% FBS). The dissociated cells were collected by centrifugation and stained with propidium iodide (PI) for flow cytometry analysis of cell viability.

### 2.3. *In Vitro* Hepatocyte Injury Model

All animal work was approved by the hospital ethics committee. Hepatocytes were isolated from C57BL/6 mice as reported previously [18]. The isolated cells were seeded into collagen-coated 6-well plates at the density of 1 × 10^4^ cells/cm^2^ and cultured using RMPI 1640 medium (Gibco) with 10% FBS. Conditioned medium of 2D cultured MSCs or 3D spheroids of MSCs were collected after 2 days of culture. To induce the hepatocyte injury* in vitro*, the culture medium of hepatocytes was replaced with fresh medium containing 3 mM CCl_4_ (Merck, Germany). After treatment with CCl_4_ for 6 h, the medium was replaced with normal medium, conditioned medium of 2D cultured MSCs, or conditioned medium of 3D spheroids of MSCs. The hepatocytes were cultured for another 24 h before lactic dehydrogenase (LDH) assays and apoptosis analysis. The LDH activity was tested according to the manufacturer's instructions, three repeats for each sample. Cell apoptosis was evaluated by Annexin V staining as indicated by the instructions. The stained hepatocytes were observed under fluorescence microscope to calculate the ratio of apoptotic cells.

### 2.4. The Mouse Model of Hepatic Fibrosis and MSC Transplantation

Adult female C57BL/6 mice were purchased from Vital River Laboratories (Beijing, China). To induce hepatic fibrosis, CCl_4_ and olive oil were mixed at 1 : 1 ratio and then injected intraperitoneally into the mice every other day [19]. After 4 weeks of treatment, 500 *μ*L of PBS, 1 × 10^6^ of 2D cultured MSCs, or 1 × 10^6^ of 3D spheroid MSCs were injected into the tail vein using a 1 mL syringes.

### 2.5. Biochemical Analysis of Serum Factors

Four weeks after MSC transplantation, the animals were sacrificed by ether anesthesia. The blood of mice was collected and the serum was isolated by centrifugation. The levels of albumin, total bilirubin (TBIL), alanine aminotransferase (ALT), and aspartate transaminase (AST) in the serum were tested using automatic biochemistry analyzer. Serum TGF-*β*1 levels were determined by ELISA. Serum hyaluronic acid (HA) levels were measured using the double-antibody sandwich chemiluminescence immunoassay. Serum alkaline phosphatase (ALP) was estimated using commercial kits (Bioassay, USA) according to instructions of manufacturer.

### 2.6. Histological Analysis

Four weeks after MSC transplantation, hepatic tissues were obtained and fixed in 4% paraformaldehyde. 4 *μ*m paraffin-embedded sections were prepared. To evaluate the hepatic fibrosis, hematoxylin and eosin (HE) and Sirius red staining were performed.

### 2.7. Reverse Transcription Polymerase Chain Reaction (RT-PCR)

Total RNA was extracted using the RNAprep Pure Cell/Bacteria Kit (TIANGEN, Beijing, China) according to the manufacturer's instructions. Reverse transcription was performed using the QuantScript RT Kit (TIANGEN) for cDNA synthesis. The PCR primers were as follows: 5′-AAA CGC AAA CAG GTT CTC AAT G-3′ (F) and 5′-CTA TGA CTG T GG TAC CTT ATA TG-3′ (R) for HGF; 5′-TCT GCA CGA GTT ACC TGT TA-3′ (F) and 5′-CAA TCT ACC AAC TCC AGG AC-3′ (R) for IGF-1; 5′-GCA CTG GCA GAA AAC AAC CT-3′ (F) and 5′-CAG GGG TGG TTA TTG CAT CT-3′ (R) for IL-6; 5′-CTC TGA CTT CAA CAG CGA CA-3′ (F) and 5′-TCT CTC TCT TCC TCT TGT GC-3′ (R) for glyceraldehyde-3-phosphate dehydrogenase (GAPDH) [20, 21]. The PCR products were detected by 2% agarose gel electrophoresis. The signals of target genes are normalized to those of the internal control.

### 2.8. Western Blotting

Hepatic tissues were homogenized and lysed in the Laemmli Sample Buffer (Bio-Rad). Proteins were quantified using the BCATM Protein Assay Kit (Thermo Scientific). For electrophoresis, 15% sodium dodecyl sulfate-polyacrylamide gel (SDS-PAGE) was prepared, and 80 *μ*g of total proteins was loaded. After electrophoresis, proteins were transferred to a PVDF membrane (Roche). The membrane was blocked with 5% nonfat dried milk (in TBST) and then incubated with primary antibodies at 4°C overnight. Unconjugated antibodies were removed by washing with TBST, and the membrane was further incubated with corresponding horseradish peroxidase-conjugated secondary antibodies. Protein bands were detected using Enhanced Chemiluminescence Reagent (Applygen). Band intensities were quantified and normalized to the internal control.

### 2.9. Statistics Analysis

All data are expressed as mean ± SD. The SPSS 17.0 software was used for statistical analysis. Student's *t*-test was used for comparison among two groups. One way ANOVA was used for comparison among multiple groups. A value of *p* < 0.05 was considered statistically significant.

## 3. Results

### 3.1. 2D Cultured MSCs and 3D Spheroids of MSCs

**Figure 1:**
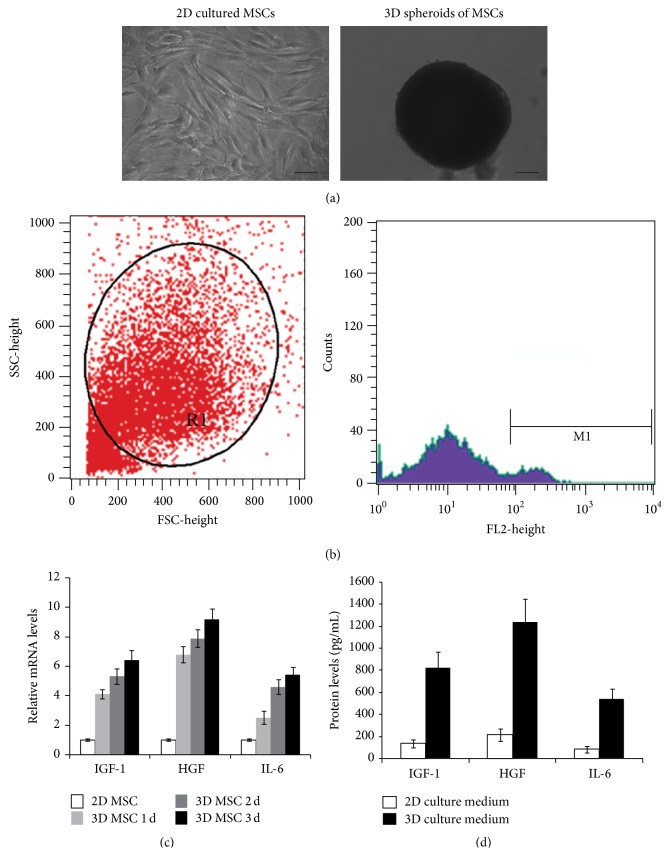
Morphology and paracrine secretions of human adipose-derived MSCs. (a) Morphology of 2D cultured MSCs and 3D spheroids of MSCs (bar = 100 *μ*m). (b) Viability of 3D spheroid-derived MSCs. 3D spheroid-derived MSCs were stained with PI and analyzed by flow cytometry, indicating that the cell viability is over 90%. (c) Paracrine secretions of MSCs. The mRNA levels of IGF-1, HGF, and IL-6 in 3D spheroids of MSCs of different days and 2D cultured MSCs were determined by RT-PCR. GAPDH was the internal control. (d) The protein levels of IGF-1, HGF, and IL-6 in the conditioned medium of 2D cultured MSCs and 3D spheroids of MSCs were determined by ELISA.

Human adipose-derived MSCs displayed fibroblast-like morphology in normal 2D culture, and the doubling time is 2–4 days. After passage 3, the morphology of cells became uniform (Figure 1(a)), which was consistent with the previous report [22]. After hanging drop culture for 24 h, MSCs formed 3D spheroids (Figure 1(a)). 3D spheroid-derived MSCs were collected by trypsin/EDTA digestion and stained with PI for flow cytometry analysis of cell viability, which indicated that the cell viability was over 90% (Figure 1(b)). After hanging drop culture for 1, 2, and 3 days, RT-PCR was used to determine the expression levels of cytokines, including insulin growth factor 1 (IGF-1), interleukin-6 (IL-6), and hepatocyte growth factor (HGF), which play important roles during inhibition of hepatic fibrosis. Compared with normally cultured MSCs, 3D spheroids of MSCs exhibited higher levels of IGF-1, IL-6, and HGF (Figure 1(c)). Furthermore, the protein levels of IGF-1, IL-6, and HGF in the conditioned medium of 3D MSCs were remarkably increased compared with that of 2D MSCs (Figure 1(d)), indicating that 3D culture might increase the therapeutic potentials of MSCs.

### 3.2. Protection of MSC-Conditioned Medium on Hepatocyte* In Vitro*

**Figure 2:**
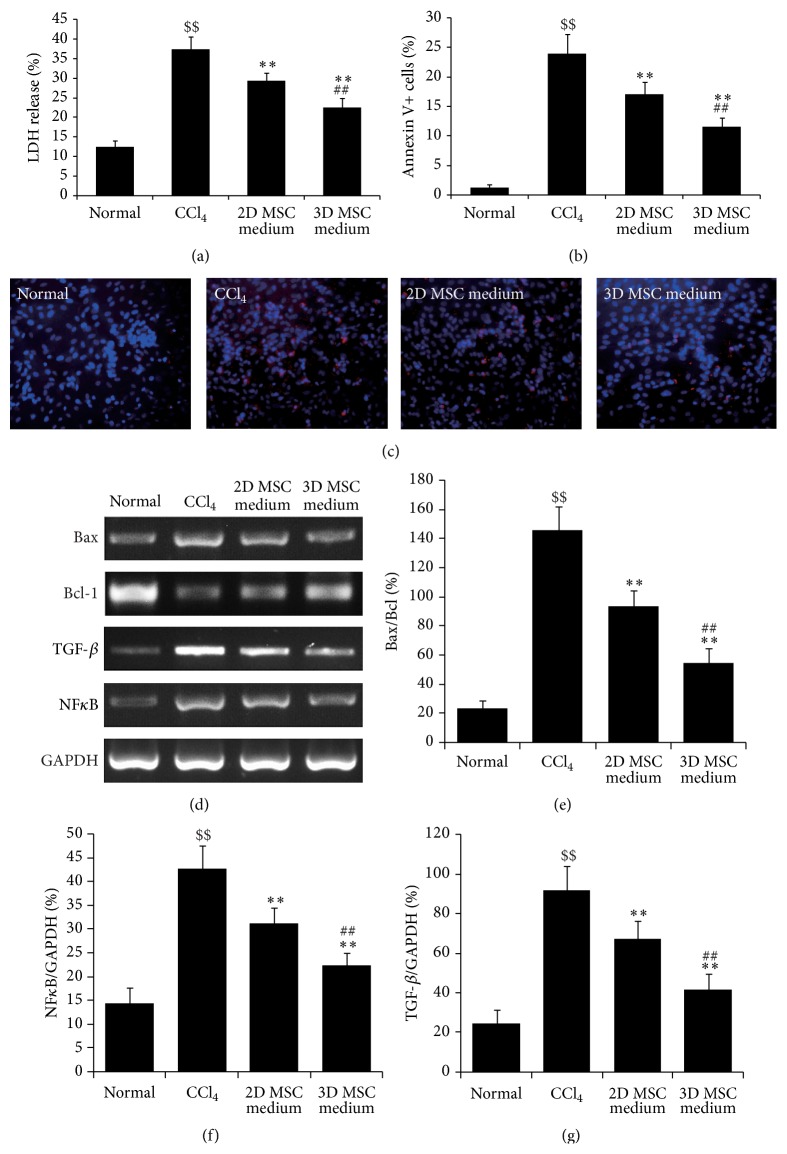
Protection of 3D spheroid-derived MSCs on hepatocyte injury* in vitro*. Hepatocytes were isolated from C57BL/6 mice and cultured in collagen-coated 6-well plates. After treatment with CCl_4_ for 6 h, the medium of cells was replaced with normal medium, conditioned medium of 2D cultured MSCs, or conditioned medium of 3D spheroids of MSCs for 24 h before LDH assays, apoptosis analysis, and RT-PCR. (a) LDH assays. (b) Statistics of Annexin V staining. (c) Images of Annexin V staining. Annexin V staining was performed to evaluate cell apoptosis (red), with DPAI for nucleus staining (blue). (d) RT-PCR. The mRNA levels of Bax, Bcl-1, NF*κ*B, and TGF-*β* were tested by RT-PCR, with GAPDH as the internal control. (e) Quantitative analysis of Bax/Bcl mRNA level. (f) Quantitative analysis of NF*κ*B mRNA level. Band intensities of NF*κ*B were normalized to those of the internal control. (g) Quantitative analysis of TGF-*β* mRNA level. Band intensities of TGF-*β* were normalized to those of the internal control. ^$$^ *p* < 0.01 compared with normal; ^*∗∗*^ *p* < 0.01 compared with CCl_4_; ^##^ *p* < 0.01 compared with 2D MSC medium.

After induction of hepatocyte injury by CCl_4_ for 6 h, mouse hepatocytes were treated with conditioned medium of 2D cultured MSCs or 3D spheroid-derived MSCs. LDH assays were performed 24 h later to evaluate the protection effects of MSC-conditioned medium on hepatocyte injury. Compared with normal hepatocytes, LDH release increased significantly after CCl_4_ stimulation (Figure 2(a)), indicating that CCl_4_ induced the hepatocyte injury. When treated with conditioned medium of 2D cultured MSCs, LDH release by hepatocytes reduced significantly (Figure 2(a)), which suggested that 2D cultured MSCs had protective roles on hepatocyte injury. More importantly, treatment with conditioned medium of 3D spheroid-derived MSCs further decreased the LDH release, compared with 2D cultured MSCs (Figure 2(a)). These results showed that 3D culture increased the protective effects of MSCs on hepatocyte injury.

Next, the apoptosis of hepatocytes was evaluated by Annexin V staining. CCl_4_ treatment induced the apoptosis of hepatocytes remarkably (Figures 2(b) and 2(c)). However, after treatment with conditioned medium of 2D cultured MSCs, the apoptotic cells reduced significantly (Figures 2(b) and 2(c)). Compared with 2D cultured MSCs, conditioned medium of 3D spheroid-derived MSCs had more remarkable effects (Figures 2(b) and 2(c)). We detected the expression levels of apoptosis-associated proteins, Bax/Bcl and NF*κ*B, as well as the fibrosis-related factor, TGF-*β*, in hepatocytes. Compared with normal hepatocytes, CCl_4_ induction increased the Bax/Bcl, NF*κ*B, and TGF-*β* expression in hepatocytes (Figures 2(d)–2(g)). Treatment with conditioned medium of 2D cultured MSCs significantly decreased the expression levels of these factors, while treatment with conditioned medium of 3D spheroid-derived MSCs had more significant effects than that of 2D cultured MSCs (Figures 2(d)–2(g)).

### 3.3. Effects of Transplanted 3D Spheroid-Derived MSCs on Liver Fibrosis

**Figure 3:**
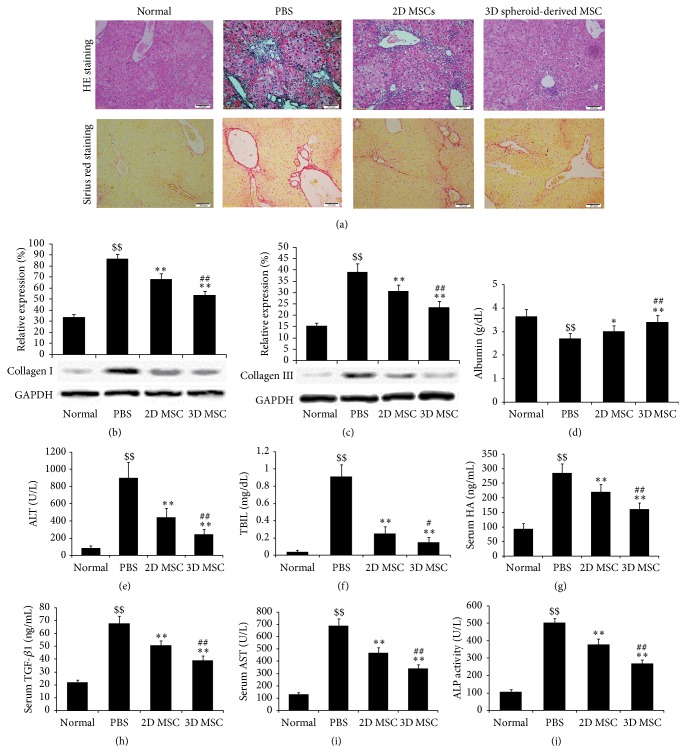
Effects of transplanted 3D spheroid-derived MSCs on hepatic fibrosis and liver function. CCl_4_ was injected intraperitoneally into adult female C57BL/6 mice to induce hepatic fibrosis. Then, 500 *μ*L of PBS, 1 × 10^6^ of 2D cultured MSCs, or 1 × 10^6^ of 3D spheroid MSCs were injected into the tail vein of hepatic fibrosis model mice. (a) HE and Sirius red staining. Four weeks after MSC transplantation, hepatic tissues were obtained and paraffin-embedded sections were prepared. HE and Sirius red staining were performed to evaluate the hepatic fibrosis (bar = 100 *μ*m). (b-c) Expression levels of fibrosis-associated factors. Four weeks after MSC transplantation, hepatic tissues were obtained and expression levels of fibrosis-associated factors (Collagen I and Collagen III) were determined by Western blotting. GAPDH was the internal control. (d–j) Analysis of serum markers associated with liver function. Four weeks after MSC transplantation, serum levels of albumin (d), ALT (e), TBIL (f), AST (i) were tested using automatic biochemistry analyzer. Serum HA (g) and TGF-*β*1 (h) levels were determined by ELISA and the double-antibody sandwich chemiluminescence immunoassay, respectively. Serum ALP activity (j) was estimated using commercial kits. ^$$^ *p* < 0.01 compared with normal; ^*∗*^ *p* < 0.05 compared with PBS and ^*∗∗*^ *p* < 0.01 compared with PBS; ^#^ *p* < 0.05 compared with 2D MSC medium and ^##^ *p* < 0.01 compared with 2D MSC medium.

Four weeks after MSC transplantation, hepatic tissues were obtained and paraffin-embedded sections were prepared. HE and Sirius red staining were performed to evaluate hepatic fibrosis. Compared with normal hepatic tissues, CCl_4_ induced hepatic fibrosis (Figure 3(a)). Transplantation of 2D cultured MSCs improved the hepatic fibrosis, while transplantation of 3D spheroid-derived MSCs exhibited more remarkable effects (Figure 3(a)). Next, the expression levels of Collagen I and Collagen III in the hepatic tissues were examined by Western blotting. Both proteins are important components of hepatic fibrosis. Compared with normal animals, the expression levels of Collagen I and Collagen III were significantly increased in hepatic fibrosis model mice (Figure 3(b)). Transplantation of 2D cultured MSCs decreased the expression levels of Collagen I and Collagen III, while transplantation of 3D spheroid-derived MSCs further reduced their expression levels (Figure 3(b)). Therefore, these results indicated that 3D spheroid-derived MSCs had stronger effects in inhibiting hepatic fibrosis than 2D cultured MSCs.

### 3.4. Effects of Transplanted 3D Spheroid-Derived MSCs on Liver Function

Four weeks after MSC transplantation, the serum markers related to liver function were analyzed. Compared with normal C57BL/6 mice, serum albumin levels were significantly reduced in hepatic fibrosis model mice (Figure 3(d)). Transplantation of 2D cultured MSCs rescued the decline of serum albumin levels (Figure 3(d)), while transplantation of 3D spheroid-derived MSCs showed more significant effects than 2D cultured MSCs (Figure 3(d)). Serum levels of ALT, TBIL, HA, TGF-*β*1, and AST were significantly increased in hepatic fibrosis model mice, compared with normal C57BL/6 mice (Figures 3(e)–3(i)). Transplantation of 2D cultured MSCs decreased the serum levels of these markers (Figures 3(e)–3(i)), while transplantation of 3D spheroid-derived MSCs exhibited more remarkable effects (Figures 3(e)–3(i)). ALP assays showed that CCL_4_ induced the increase of serum ALP activity in model mice compared with normal C57BL/6 mice (Figure 3(j)). Transplantation of 2D cultured MSCs decreased the serum ALP activity (Figure 3(j)), while transplantation of 3D spheroid-derived MSCs further decreased the serum ALP activity (Figure 3(j)). Altogether, these results showed that 3D spheroid-derived MSCs had stronger effects in ameliorating liver function than 2D cultured MSCs when transplanted into the hepatic fibrosis model mice.

## 4. Discussion

MSC-based therapy is promising for hepatic fibrosis treatment, and paracrine secretions act as an important mechanism. However, paracrine secretions are limited for normally cultured MSCs, impairing their therapeutic effects. 3D culture was reported to enhance the therapeutic potentials of MSCs [13, 14, 17]. In this study, we compared therapeutic effects of 2D cultured MSCs and 3D spheroids of MSCs for hepatic fibrosis and showed that transplanted 3D spheroids were more effective in improving liver function and ameliorating hepatic fibrosis.

Compared with 2D cultured MSCs, 3D spheroids showed higher expression levels of antifibrotic genes, including IL-6, IGF-1, and HGF.* In vitro* studies indicated conditioned medium of 3D cultured MSCs protected hepatocytes from cell injury and apoptosis more effectively than 2D cultured cells. Furthermore, conditioned medium of 3D cultured MSCs downregulated the expression of fibrosis-related genes (e.g., NF*κ*B and TGF-*β*) in hepatocytes more remarkably. These results suggested that paracrine secretions of beneficial cytokines played an important role in protecting hepatocytes from cell damage and fibrosis formation.

Consistent with* in vitro* experiments, when transplanted into model mice with hepatic fibrosis, 3D spheroids of MSCs were more beneficial in ameliorating hepatic fibrosis than 2D cultured cells, as shown by HE and Sirius red staining. Accordingly, the levels of Collagen I and Collagen III, two major components of fibrosis, were also significantly reduced after 3D spheroid transplantation. More importantly, the model mice which accepted 3D spheroid transplantation showed improved liver function, as indicated by a variety of serum markers, including albumin, ALT, TBIL, HA, TGF-*β*1, AST, and ALP. These results indicated that 3D spheroids of MSCs are more effective in improving liver function and ameliorating hepatic fibrosis than 2D cultured cells.

Various strategies have been investigated to enhance the therapeutic effects of MSCs, including gene modification, hypoxia preconditioning, and cotransplantation with adjuvant. Genetically modified MSCs show enhanced paracrine secretions [23] or stronger survival [24], but gene modification has the risk to activate oncogenes. Cotransplantation with adjuvant such as chitosan hydrogel facilitates the retention and survival of stem cells but fails to stimulate their paracrine secretions [25, 26]. Hypoxia preconditioning not only increased the survival of transplanted MSCs, but also activated the expression of prosurvival and angiogenic factors [27–31]. 3D culture promoted differentiation of MSCs [11, 12] and enhanced their paracrine secretions of beneficial cytokines, including anti-inflammatory, angiogenic, and antiapoptotic factors [13]. In this study, our results indicated that the expression of antifibrotic factors is also increased in 3D cultured MSCs.

In conclusion, we showed that 3D spheroids of MSCs were more beneficial in improving liver function and ameliorating hepatic fibrosis than 2D cultured cells. The 3D culture strategy improved the therapeutic effects of MSCs and might be promising for treatment of hepatic fibrosis.
